# Health Tract No. 333

**Published:** 1868-09

**Authors:** 


HEALTH TRACT No. 333.

### CHILDREN'S TEETH.

The first tooth appears in six months, in the third year all are cut," between the seventh and twenty-first year all the permanent teeth have made their appearance ; the value of the latter depends greatly on the care taken of the first set ; and as the looks, health and happiness are all materially modified by good teeth , intelligent and affectionate parents will look to the teeth of their children as early as the third year, when, instead of being allowed to eat meat, they should be mainly fed on fruits, vegetables and bread made of wheat, corn or rye, ground coarsely, using the entire product, bran and all, because in the bran, is found almost exclusively, the solid material which is to make the bone or body of the tooth and its covering, called the enamel.

The child should be taught at five, to dampen the brush in water every morning, rub it over a cake of castile soap and then brush the teeth well, inside and out, front and rear ; until, with the aid only of the saliva, the mouth is full of soap-suds ; then rinse with tepid water, twirling the brush sideways over the back part of the tongue, so as to cleanse it fully of the soap and leave a good taste ; after each meal, the mouth should be well rinsed with tepid water, as also the last thing on retiring ; the mouth maintains a temperature of ninety-eight degrees ; hence, if any food lodges about or between the teeth, it begins to rot very soon, giving out an acid which immediately begins to eat into the tooth, preparatory to an early decay ; if solid particles are observed to lodge between the teeth, the child should be taught to use a very thin quill to dislodge it ; but not without, for the more a quill is used the greater space between the teeth, which is a misfortune, as it necessitates the use of a toothpick for all after life, consuming a great deal of valuable time. It is a wicked blunder to advise that a silk thread should be sawed between the teeth after eating ; nature intended the teeth to grow so close together that nothing could get between them ; it is a bad practice, except in very rare cases, to remove the stump of the first teeth ; let them be displaced by nature's own process ; that dentist is an ignoramus, who advises a sound tooth to be drawn to " give more room " for the others, thus preventing that natural expansion of the jaw, which gives " character " to the face, and greater power of mastication, an essential element of an easy and healthful digestion of the food. A clean tooth does not decay. Acids, sour fruits, always injure the teeth instantly ; sweets, never do ; without them, children would die, hence their insatiable instincts for sugar. If a tooth powder was never used, the teeth would not be so white ; but kept perfectly clean, would last for life.

